# Alteration of cGAS-STING signaling pathway components in the mouse cortex and hippocampus during healthy brain aging

**DOI:** 10.3389/fnagi.2024.1429005

**Published:** 2024-08-01

**Authors:** Sergio Passarella, Shananthan Kethiswaran, Karina Brandes, I-Chin Tsai, Kristin Cebulski, Andrea Kröger, Daniela C. Dieterich, Peter Landgraf

**Affiliations:** ^1^Institute for Pharmacology and Toxicology, Otto-von-Guericke University Magdeburg, Magdeburg, Germany; ^2^Institute of Medical Microbiology and Hospital Hygiene, Molecular Microbiology, Health Campus Immunology, Infectiology and Inflammation, Otto-von-Guericke University Magdeburg, Magdeburg, Germany; ^3^Innate Immunity and Infection, Helmholtz Centre for Infection Research (HZI), Braunschweig, Germany; ^4^Center for Behavioral Brain Sciences, Magdeburg, Germany

**Keywords:** cGAS-STING pathway, brain aging, autophagy, senescence, cortex, hippocampus

## Abstract

The cGAS-STING pathway is a pivotal element of the innate immune system, recognizing cytosolic DNA to initiate the production of type I interferons and pro-inflammatory cytokines. This study investigates the alterations of the cGAS-STING signaling components in the cortex and hippocampus of mice aged 24 and 108 weeks. In the cortex of old mice, an increase in the dsDNA sensor protein cGAS and its product 2′3′-cGAMP was observed, without corresponding activation of downstream signaling, suggesting an uncoupling of cGAS activity from STING activation. This phenomenon may be attributed to increased dsDNA concentrations in the EC neurons, potentially arising from nuclear DNA damage. Contrastingly, the hippocampus did not exhibit increased cGAS activity with aging, but there was a notable elevation in STING levels, particularly in microglia, neurons and astrocytes. This increase in STING did not correlate with enhanced IRF3 activation, indicating that brain inflammation induced by the cGAS-STING pathway may manifest extremely late in the aging process. Furthermore, we highlight the role of autophagy and its interplay with the cGAS-STING pathway, with evidence of autophagy dysfunction in aged hippocampal neurons leading to STING accumulation. These findings underscore the complexity of the cGAS-STING pathway’s involvement in brain aging, with regional variations in activity and potential implications for neurodegenerative diseases.

## Introduction

The cGAS (cyclic GMP-AMP synthase)−STING (stimulator of interferon genes) pathway is a key mechanism of innate immunity that detects the presence of cytosolic double stranded DNA and triggers the production of type I interferons and pro-inflammatory cytokines (Li et al., 2013). Cytosolic DNA can originate from various sources, such as viral or bacterial infections, damage of nuclear DNA, or mitochondrial leakage (Glück et al., 2017; Takahashi et al., 2018). The initial components of cGAS-STING pathway are the cytosolic DNA sensor cGAS and the endoplasmic reticulum (ER)-resident adaptor protein STING (Ishikawa and Barber, 2008; Ishikawa et al., 2009; Sun et al., 2013). Upon binding to cytosolic DNA, cGAS catalyzes the synthesis of a cyclic dinucleotide, 2′3′-cyclic GMP-AMP (2′3′-cGAMP), which acts as a second messenger and binds to STING (Ishikawa et al., 2009; Sun et al., 2013). After binding, STING undergoes a conformational change and translocates from the ER to perinuclear vesicles, where it interacts with and activates downstream kinases, such as TBK1 and IKK, which in turn phosphorylate and activate the transcription factors IRF3 and/or NF-κB (Li et al., 2013; Sun et al., 2013). These transcription factors translocate to the nucleus and induce the expression of type I interferons and pro-inflammatory cytokines or chemokines, such as TNF-α, IL-6, and IL-1β (Li et al., 2013; Sun et al., 2013). Subsequently, these cytokines can trigger and amplify inflammatory responses, and modulates the adaptive immune system by activating dendritic cells, T cells, and B cells. Thus, the cGAS-STING pathway plays an essential role in defending against viral and bacterial infections, as well as in modulating cellular homeostasis and stress responses (Li et al., 2013; West et al., 2015). However, recent studies have revealed that the cGAS-STING pathway can also contribute to chronic inflammation and tissue damage in various pathological conditions, such as autoimmune diseases, cancer, and neurodegeneration (Ahn et al., 2014, 2018; Woo et al., 2014; West et al., 2015; Abdullah et al., 2018; Choi et al., 2020; Hinkle et al., 2022; Skopelja-Gardner et al., 2022; Zhao J. et al., 2022; Gulen et al., 2023; Xie et al., 2023). In these conditions, the cGAS-STING pathway can be activated by aberrant accumulation of self-DNA in the cytosol, which can result from defective DNA repair or mitochondrial dysfunction (West et al., 2015; Skopelja-Gardner et al., 2022). The sustained activation of the cGAS-STING pathway can lead to a vicious cycle of inflammation and tissue damage, which further exacerbates the disease progression and severity (Skopelja-Gardner et al., 2022; Zhao J. et al., 2022).

One of the emerging aspects of the cGAS-STING pathway is its involvement in the regulation of aging and age-related diseases (Gulen et al., 2023; Zheng et al., 2023; Jiménez-Loygorri et al., 2024). Aging is characterized by a progressive decline in physiological functions and an increased susceptibility to various disorders, such as cardiovascular diseases, diabetes, and neurodegenerative diseases. Aging is also associated with a low-grade chronic inflammation, termed inflammaging, which is driven by multiple factors, such as cellular senescence, mitochondrial dysfunction, and genomic instability (Franceschi et al., 2017, 2018; Ferrucci and Fabbri, 2018).

Recent evidence suggests that the cGAS-STING pathway is a critical mediator of inflammagin and neurodegeneration in the central nervous system (CNS) (Paul et al., 2021; Hinkle et al., 2022; Gulen et al., 2023; Xie et al., 2023). Several studies have shown that the cGAS-STING pathway is activated in the brain of aged mice and humans, as well as in mouse models of Alzheimer’s disease (AD), Parkinson’s disease (PD), and amyotrophic lateral sclerosis (ALS) (Yu et al., 2020; Hinkle et al., 2022; Tan et al., 2022; Xie et al., 2023). Furthermore, recent studies have suggested that the cGAS-STING pathway may modulate autophagy in different cell types and under different conditions (Prabakaran et al., 2018; Gui et al., 2019; Sharma et al., 2020). For instance, cGAS-STING activation can induce autophagy through WIPI2 and ATG5, independent of TBK1 and IRF3, in response to DNA damage or viral infection (Liu et al., 2019; Wan et al., 2023). Conversely, cGAS-STING inhibition can impair autophagy by reducing the expression of autophagy-related genes, such as BECN1 and ATG7, in cancer cells (Liu et al., 2019; Zheng et al., 2023). Furthermore, p62, an important cargo-protein involved in the formation of the autophagosome, has been found that it can attenuate the cGAS-STING activity via degradation of STING (Prabakaran et al., 2018). Research conducted on herpes simplex virus type 1 (HSV-1)-infected mouse revealed that the cGAS–STING pathway was the major mechanism by which HSV-1 infection enhanced LC3 puncta production and autophagic flux (Reinert et al., 2016). Additionally, it was proposed that the cGAS–STING system’s ancient and highly conserved role in autophagy precedes the formation of the type-I interferon pathway in vertebrates. When stimulated by 2′3′-cGAMP, STING promotes autophagy in the sea anemone (*Nematostella vectensis*), but not interferon, suggesting that parts of the autophagy machinery are closely interconnected with the cGAS–STING system. Moreover, cGAS-STING pathway and autophagy can interact in a feedback loop, as autophagy can degrade cGAS and STING, thereby limiting their activation and signaling. However, the crosstalk between cGAS-STING signaling and autophagy in the brain and its implications for brain aging remain unclear.

Here, we describe how components of the cGAS-STING pathway are modulated during mice brain aging using 24, and 108-week-old animals. We focus our attention on specific brain areas known to be specifically affected during aging like the entorhinal cortex (EC) and hippocampus that are involved in memory, navigation, and spatial processing (Knierim, 2015; Rechnitz and Derdikman, 2023). Furthermore, EC and hippocampus are also one of the first areas to be affected by AD, a neurodegenerative disorder that causes progressive memory loss and cognitive decline (Igarashi, 2023). Our results provide evidence that in normal brain aging, components of the cGAS-STING pathway and autophagy are differently altered in the hippocampus and EC of mice.

## Materials and methods

### Animals

The study was carried out in accordance with the recommendations of the National Committee for the Protection of Animals Used for Scientific Purposes of the Federal Republic of Germany and European regulations for ethical care and use of laboratory animals (2010/63/EU). The experiments were approved by the local Ethics Commission of the Federal State of Saxony-Anhalt (42502-2-1507 Uni MD and 42502-2-1578 Uni MD). Male and female C57BL/6J wild type (WT) mice (*Mus musculus*) were housed under controlled laboratory conditions at constant temperature (20 ± 2°C) and air humidity (55–60%). 12 h light–dark cycles were used (lights on at 6:00 a.m.). Animals had access to commercial food pellets and tap water *ad libitum*. The health status of animals was monitored regularly.

### Immunoblotting

Cortex brain tissues were homogenized using a Potter S (Sartorius, Göttingen, Germany) in 1x PBS (ratio 10 ml/1 g), pH 7.4, containing EDTA-free complete™ protease inhibitor cocktail (Roche, Basel, Switzerland), phosphatase inhibitor (PhosSTOP™, Roche, Basel, Switzerland) and Benzonase nuclease (Merck). Then the homogenates were diluted with SDS and boiled at 95°C for 5 min cooled on ice and stored at −20°C. The respective protein concentrations were determined using the amido black assay according to Badin and Herve (1965). The samples were run on 5–20% SDS-polyacrylamide gels using a Hoefer™ Mighty Small System (Fisher Scientific). Proteins were separated at a current of 12 mA per gel and a constant temperature of 4°C. Then, proteins were transferred with 10 μg/lane or 20 μg/lane and spotted onto nitrocellulose membrane (Protran BA85, 0.22 μm, Li-Cor Biosciences). Subsequently, the membranes were stained with Ponceau staining solution (0.5% Ponceau S, 3% acetic acid in ddH_2_O) for 10 min and after destaining the membranes were incubated at 4°C overnight with primary antibodies in the respective concentration using 1xTBS containing 0.1% Tween 20 including the following antibodies: LC3 A/B (Cat. No. #4108S, dilution 1:1000 in blocking solution, Cell Signaling Technology), p62 (Cat. No. #5114, dilution 1:1000 in blocking solution, Cell Signaling Technology), TOM20 (Cat. No. #42406, dilution 1:1000 in blocking solution, Cell Signaling Technology), Histone H2AX (Cat. No. 10856-1-AP, dilution 1:1000 in blocking solution, Proteintech), cGAS (Cat. No. #31659, dilution 1:1000 in blocking solution, Cell Signaling Technology), STING (Cat. No. 19851-1-AP, dilution 1:1000 in blocking solution, Proteintech), TBK1 (Cat. No. #29047, dilution 1:1000 in blocking solution, Cell Signaling Technology), pTBK1 (Ser172) (Cat. No. #5483, dilution 1:1000 in blocking solution, Cell Signaling Technology), IRF3 (Cat. No. ab68481, dilution 1:1000 in blocking solution, Abcam, Cambridge, MA), pIRF3 (Ser396) (Cat. No. PA5-38285, dilution 1:1000 in blocking solution, Invitrogen). HRP-conjugated secondary antibodies (1:7500) were used for quantitative immunoblotting. All Imaging and quantifications were done using Image Studio™ software (LI-COR^®^ Biosciences GmbH, version 5.2.5) and LI-COR OdysseyFC (LI-COR). Normalization was carried out on the control group and related to the corresponding Actin (monoclonal mouse, Cat. No. #3700, dilution 1:2000 in blocking solution, Cell Signaling Technology and monoclonal rabbit, Cat No. #8457, dilution 1:2000 in blocking solution, Cell Signaling Technology) values. At least two technical replicates were done for all quantifications.

### Immunohistochemical stainings

For immunohistochemical staining, mice aged 24, and 108-week-old were anesthetized with an intraperitoneal injection of ketamine solution (100 mg/kg). Mice were fixed on a Styrofoam plate, the thorax opened and then transcardially perfused, first with PBS to remove blood from brain tissue and then with 4% paraformaldehyde (PFA, Serva). Subsequently, mice were decapitated, the brains removed and incubated overnight at room temperature in 4% PFA with 4% Sucrose, then submerged in 30% (w/v) sucrose for two days and finally embedded in tissue freezing medium (Leica Microsystems) and frozen at −70 °C. Later, brains were cut into 20 μm thick frontal slices using the Leica CM3050 S cryostat (Leica). The slices were stored until further processing at 4°C in a TPBS solution containing 0.01% sodium azide.

All incubation and washing steps were done under constant gentle shaking. Before staining, slices were washed three times in TPBS (0.12% Tris, 0.9% NaCl, 0.025% NaH2PO4). Permeabilization of the sections was achieved by 30 min incubation in a TPBS/ethanol mixture (1:1). Subsequently, slices were washed with TPBS three times for 5 min each and stained with the primary antibody in TPBS, containing 1% horse serum and 0.25% Triton X-100, overnight in the dark at 4°C. The following antibodies were used: guinea pig anti-NeuN (Cat. No. 266004 dilution 1:300 in blocking solution, Synaptic Systems), chicken anti-GFAP (Cat. No. ab4674, dilution 1:300 in blocking solution, Abcam, Cambridge, MA), goat anti-Iba1 (Cat. No. ab5076, dilution 1:300 in blocking solution, Abcam, Cambridge, MA), rabbit anti-STING (Cat. No. 19851-1-AP, dilution 1:100 in blocking solution, Proteintech), mouse anti-p62 (Cat. No. ab56416, dilution 1:100 in blocking solution, Abcam, Cambridge, MA), mouse anti-dsDNA (Cat No. ab27156, 1:100 in blocking solution, Abcam, Cambridge, MA). Slices were washed four times with TPBS including 0.3% Triton X-100 and incubated with respective secondary antibodies that are coupled with Alexa Fluor dyes (Abcam or Jackson Immunoresearch Europe) in TPBS, 1% horse serum and 0.25% Triton X-100 for 4 h at 4°C. Slices were then dried on gelatin-covered slides (Superfrost^®^ 26 x 76 mm, Roth) for 30 min at 37°C. The slides were each incubated for 2 min in 70, 80, 90, 100% isopropanol and twice for 5 min in Roti^®^Histol (Roth). Finally, the slides were dried, mounted with Neo-Mount™ (Millipore) and covered with coverslips (Menzel™ 11911998).

### Microscopy and image processing

Immunohistochemistry staining were obtained via confocal laser scanning microscopy (Zeiss Axio Oberserver Z.1 confocal microscope with the confocal LSM 710 unit and Zen Black Edition software; Carl-Zeiss Jena). Cells were selected from slices of four to six mice with uniform sex distribution. All images were taken as 8-bit in Z-stacks with an EC Plan Neofluar 20x except for the magnifications where a Plan-Apochromat 63 × numerical aperture 1.4 oil DIC M27 immersion objective was used. The confocal microscope images were processed as maximum intensity projections confocal pictures (425.1x425.1 μm – 1024x1024 pixels) and analyzed in FIJI (version 2.0). For intensity analysis, the intensity values of each picture were related to the respective mean intensity of the control group. Identical exposure times for quantification of all cortical and hippocampal regions analyzed were used. The FIJI plugin called JACoP with Pearson coefficient evaluation was used for all colocalization analyses (Bolte and Cordelières, 2006). For the evaluation of the number of NeuN^+^, STING^+^, IBA1^+^ cells and p62 puncta on the NeuN mask at cortical and hippocampal levels, the FIJI’s plugin was used to analyze particles with a defined threshold that was maintained throughout the analysis.

### 2′3′-cGAMP ELISA

Mouse tissues were lysed in Pierce RIPA Buffer containing EDTA-free complete™ protease inhibitor cocktail (Roche, Basel, Switzerland) and phosphatase inhibitor (PhosSTOP™, Roche, Basel, Switzerland) using a potter machine (Sartorius, Göttingen, Germany). The respective protein concentrations were determined using the amido black assay according to Badin & Herve (Badin and Herve, 1965). For measuring 2′3′-cGAMP, a competitive ELISA kit (Item No 501700, Cayman Chemical) was used following the manufacturer’s instructions and analyzed at a wavelength of 450 nm using an Infinite F50 (Tecan) photometer.

### RNA isolation and qRT-PCR

For quantitative RT-PCR of RNA expression levels, mouse organs were homogenized in peqGOLD TriFast (PeqLab, #30-2010) using the homogenizer Fast Prep 24 (MP). RNA was isolated according to the manufacturer’s instruction. cDNA synthesis from RNA was done with the M-MLV reverse transcriptase kit (Invitrogen) and quantitative qRT-PCR was carried out by using KAPA SYBR FAST Universal Master Mix (PeqLab). The following murine primers were used for qPCR: pan-IFN-α (forward primer 3′-CTA GAC TCA TTC TGC AAT G-5′, reverse primer 3′-TCC TCA CAG CCA GCA GG-5′), IFNb (forward primer 3′-CTT CTC CGT CAT CTC CAT AGG G-5′, reverse primer 3′-CAC AGC CCT CTC CAT CAA CT-5′), ISG-54 (IFIT-2) (forward primer 3′- CAC CTT CGG TAT GGC AAC TT -5′, reverse primer 3′- GCA AGG CCT CAG AAT CAG AC-5′). All samples were measured by Light Cycler 480 II (Roche), normalized to gene expression of β-actin and calculated by the ΔΔCT method.

### Quantification and statistical analysis

Data are shown as mean ± SEM. Two-tailed Student’s *t* test was used to determine statistical differences among the two groups (24 and 108-week-old) for Western blot, ELISA, RT-PCR and images data. Differences between mean values *P* < 0.05 were considered statistically significant. All present data were analyzed and graphed with Prism 8 (GraphPad Software Inc. La Jolla, CA, USA).

## Results

### cGAS and 2,3-cGAMP are upregulated in the aged cortex, but do not trigger inflammation

**FIGURE 1:**
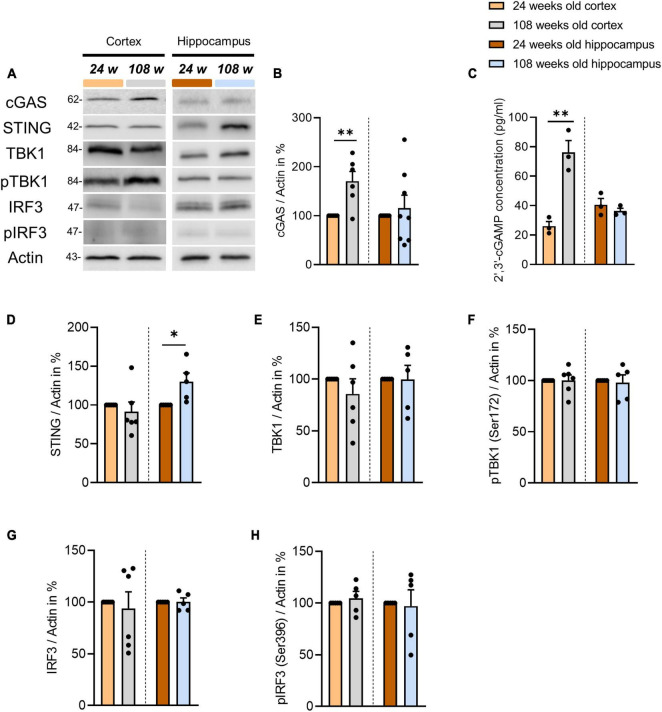
Alterations of cGAS-STING pathway components in the cortex and hippocampus of the aging mouse brain. **(A)** Representative blots of proteins involved in cGAS-STING signaling in cortical and hippocampal tissue using Actin as an internal control. **(B,D–H)** Determination of selected protein levels related to the cGAS-STING pathway using WB. Relative quantities were normalized to Actin. Values are the mean of three technical replicates in each group from 5 to 6 independent experiments and biological replicates for immunoblotting analysis. **(C)** 2′3′-cGAMP level were measured by ELISA in the cortical and hippocampal tissues of 24- and 108-weeks-old mice using three animals per group. All data are presented as the mean ± SEM. Symbols for *P*-values used in the figures: *P < 0.05, **P < 0.01. *P*-values were calculated using a two-tailed Student’s *t*-test.

To study the role of the cGAS-STING pathway in brain homeostasis during aging, we analyzed C57BL/6J mice from two age groups: 24 weeks (young) and 108 weeks of age (old). The selected regions were the cortex and hippocampus i.e., the areas that have been shown to be most affected by cognitive aging processes (Rodrigue and Raz, 2004; Du et al., 2006). First, we examined the protein levels of all protagonists involved in the cGAS-STING signaling in cortical tissue and we found that cGAS is the only protein that increases significantly in aged mice (Figures 1A, B). Next, we analyzed the amount of 2′3′-cGAMP, the product of cGAS activity, in cortical tissue using ELISA to determine whether the age-dependent elevation of cGAS levels correlates with an increase in its activity. We found that 2′3′-cGAMP was indeed statistically increased in 108-week-old mice (Figure 1C). 2′3′-cGAMP is responsible for the activation of STING, which in turn leads to TBK1 and IRF3 phosphorylation, which triggers inflammatory responses. Therefore, we examined the phosphorylation of TBK1 and IRF3 using WB but we found no differences between the experimental groups analyzed (Figures 1E–H). Finally, we checked selected genes activated by the phosphorylated transcription factor IRF3, such as ISG54, IFN-α, and IFN-β, and found no significant differences between the two age groups analyzed (Supplementary Figures 1A–C). In a next step, we focused on the cGAS-STING pathway components in the hippocampus. Using WB’s we examined the protein expression of cGAS-STING signaling actors in order to identify plausible mechanisms underpinning the pathway function in the hippocampus. In contrast to the cortex, the levels of cGAS and 2′3′-cGAMP did not change with age (Figures 1A–C). However, similar to the cortex, the age-dependent unchanged level of TBK1 and IRF3 phosphorylation did not indicate a change in the inflammatory response (Figures 1A, E–H). Interestingly, we observed an increase in STING protein concentration in the hippocampus of aged mice, in contrast to the cortex (Figures 1A, D).

**FIGURE 2:**
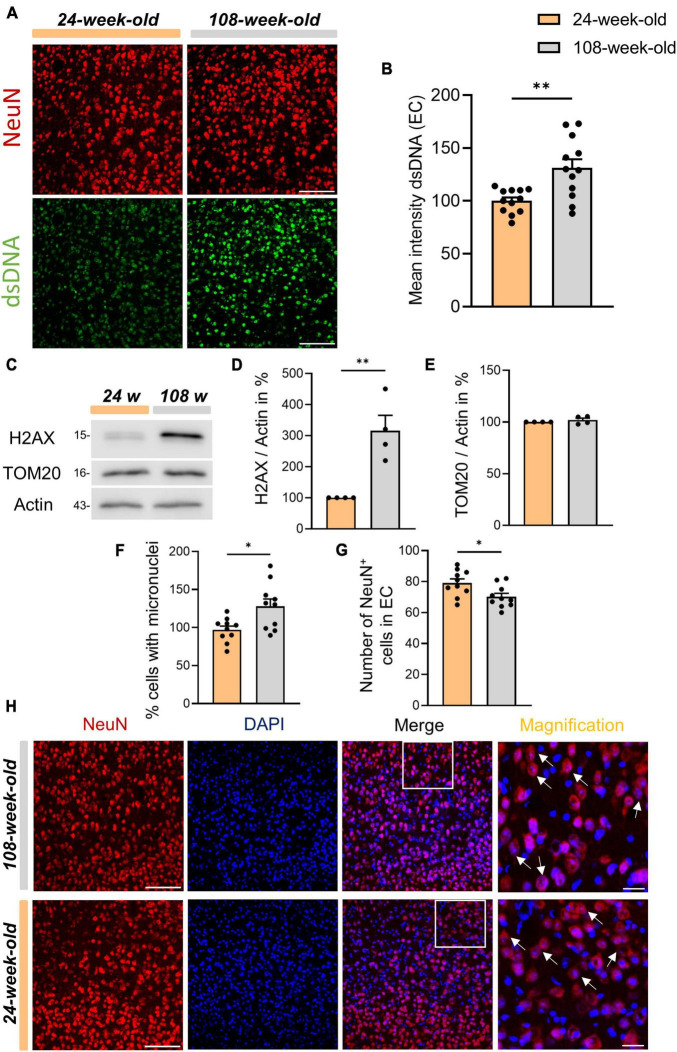
Increased dsDNA accumulation in micronuclei of cortical neurons in old mice. **(A)** Anti-dsDNA (green) and NeuN (red) immunostaining of EC from young and old mice. **(B)** Evaluation of dsDNA intensity on EC, scale bar = 100 μm. **(C)** Representative Western-blots of H2AX, TOM20 and Actin as an internal control. **(D,E)** Quantification of protein levels of H2AX and TOM20. Protein levels are normalized to the loading control (Actin). Values are the mean of three technical replicates in each group from four independent experiments for immunoblotting analysis. **(F)** Evaluation of the relative amounts of NeuN^+^ cells with micronuclei. **(G)** Quantification of the number of NeuN^+^ cells in EC. **(H)** Immunofluorescence staining of DAPI and NeuN in the EC of 24- and 108-week-old mice, scale bar = 100 μm. From the merged image, a distinctive area was taken and magnified. In the magnification, arrows were inserted to highlight the micronuclei present within the neuronal soma. All data are presented as the mean ± SEM. Symbols for *P*-values used in the figures: *P < 0.05, **P < 0.01. *P*-values were calculated using a two-tailed Student’s *t*-test.

Next, we asked the question, why exclusively cGAS and its product 2′3′-cGAMP were upregulated in the cortices of aged mice. Since cGAS is directly involved in the DNA damage repair process, we assessed the amount of dsDNA and the H2AX protein as a marker for DNA damage (Liu et al., 2018; Jiang et al., 2019). First, we quantified the dsDNA at the cortical level in fluorescence-immunohistochemistry using the EC, one of the cortical regions most impacted by aging events (Nassif et al., 2022). Using NeuN as a marker of the neuronal soma, we were able to see a significant increase in dsDNA in the EC, particularly in neurons (Figures 2A, B). Furthermore, we were able to determine that the cortices of 108-week-old mice had significantly more DNA damage than 24-week-old animals (Figures 2C, D). In contrast, in hippocampal tissues of the DG and CA1 region, as opposed to the cortex, we found no increase in dsDNA or H2AX (Supplementary Figures 1D–I).

Subsequently, in order to determine the source of cytosolic dsDNA accumulation in cortical neurons, we used TOM20 to analyze mitochondrial homeostasis and found no evidence of aging-related alterations (Figures 2C, E). Thus, we analyzed the content of EC micronuclei in neurons. In line with earlier studies, we found that the ECs of 108-week-old mice had more micronuclei in neurons when compared to 24 week-old mice, as shown by immunohistochemistry using DAPI and NeuN (Figures 2F, G; Migliore et al., 2011; Andriani et al., 2017; Shepherd et al., 2018). Moreover, we observed a marked decline in the quantity of NeuN-positive cells in the EC with aging that might be attributed to a higher incidence of neuronal death in old mice aged 108 weeks (Figure 2H). In addition, to analyze autophagic processes in the cerebral cortex during aging we evaluated the main autophagic markers p62 and LC3 by WB which showed no changes between the two age groups. At the same time, the signal intensity analysis of p62 in immunohistochemistry in EC also reviled no changes between the age groups of 24 weeks and 108 weeks (Supplementary Figures 2A–F). Overall, our results, on the one hand, confirm the function of cGAS in identifying dsDNA, which is mainly derived from micronuclei in the cytoplasm of cortical neurons, but on the other hand, this process does not seem to be associated with the induction of an inflammatory response. Furthermore, we demonstrated that the protein amounts of the key players in the cGAS-STING pathway vary during aging according to the region of interest showing differences between hippocampus and cortex.

### STING accumulates in neurons and astrocytes of the hippocampus during brain aging

The tendency to lose perception of time and place is a grave sign of aging. Alterations in the entorhinal cortex and the hippocampal regions of the brain’s medial temporal lobe are responsible for this symptoms (Mulder et al., 2016; Roberts and Allen, 2016). Therefore, after analyzing the data in the EC, we focused on understanding the reason for the increase of STING present in the hippocampal samples of 108-week-old animals compared with the 24-week-old group (Figure 1D).

**FIGURE 3:**
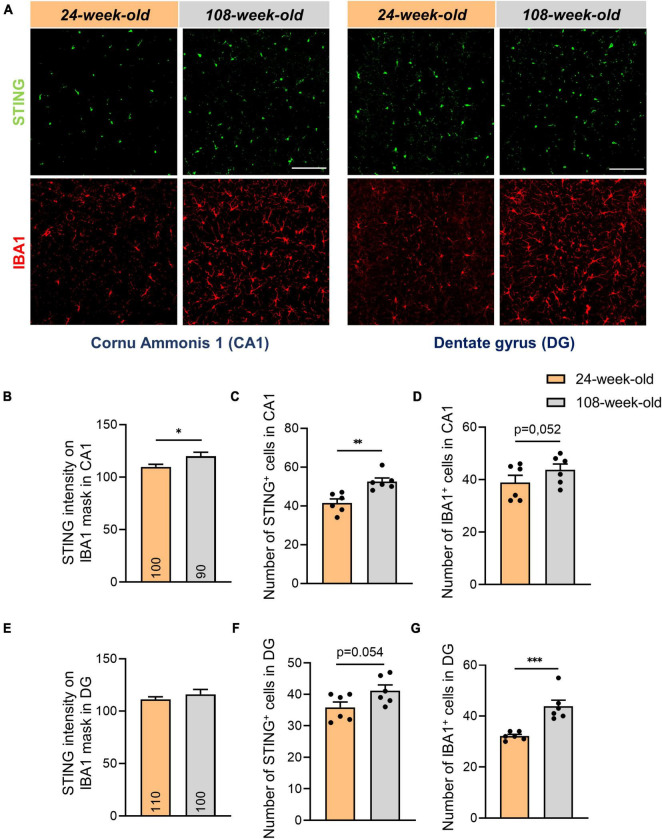
Increased number of microglial- and STING positive cells in hippocampal areas of 108-week-old mice. **(A)** Representative images showing STING and IBA1 staining in CA1 and DG hippocampal slices of young and old mouse brains, scale bar = 100 μm. **(B,E)** Quantification of the STING intensity on IBA1 mask in CA1 and DG regions. **(C,D,F,G)** Evaluation of number of IBA1^+^ and STING^+^ cells in DG and CA1. Data were collected with a *N* = 4–6 biological replicates. All data are presented as the mean ± SEM. Symbols for *P* values used in the figures: *P < 0.05, **P < 0.01, ***P < 0.001. *P*-values were calculated using a two-tailed Student’s *t*-test.

Since microglia in the brain express STING more than other cell types (Supplementary Figure 3A), we examined the amount of IBA1^+^ cells in DG and CA1 to ascertain the reason of the increase of the STING protein in the hippocampus. In contrast to the EC (Supplementary Figure 3B) we observed a substantial aging-related increase in IBA1^+^ cells in these regions while we did not notice differences in the signal intensity of the same protein (Figures 3A, D, G, Supplementary Figures 3F, G). We also showed that when microglia aged, the STING signal intensity increased in CA1, whereas it wasn’t affected in the EC and DG (Figures 3B, E, Supplementary Figures 3C–E). In this context we questioned whether there would be a higher cGAS-STING related inflammation in the hippocampal region of the brain due to the increase in number of microglia. Similar to EC, we also did not observe an increase in the expression of genes dependent on IRF3 phosphorylation such as ISG54, IFN-α in the hippocampus of 108-week-old mice (Supplementary Figures 3M–O).

**FIGURE 4:**
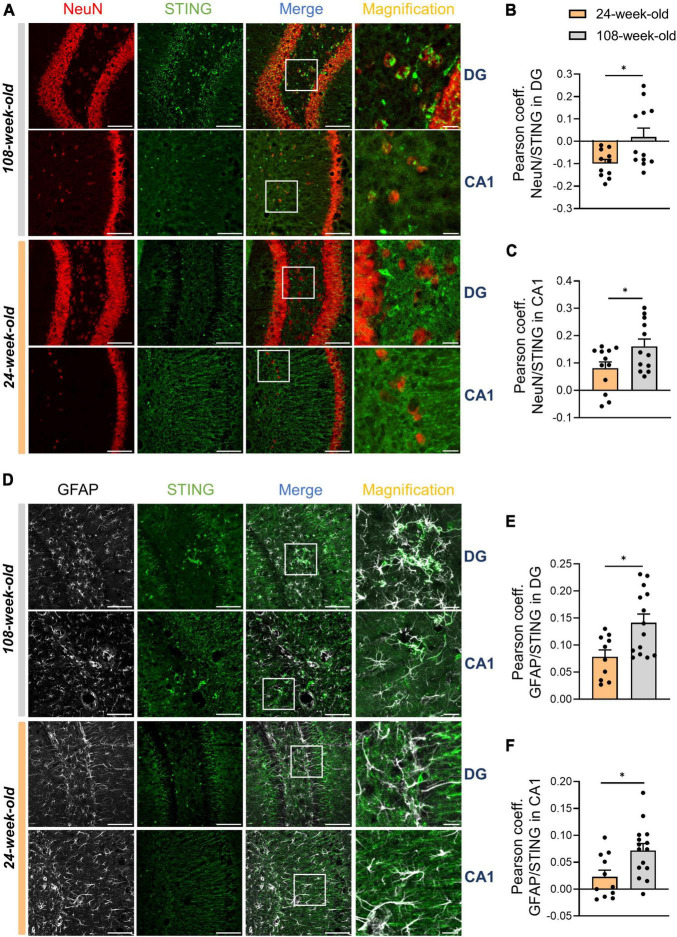
STING increases its expression on neurons and astrocytes during hippocampal aging. **(A–C)** Representative immunostainings for STING (green) and NeuN (red) (scale bar = 100 μm, magnification: scale bar = 20 μm) for 108-week-old and 24-weel-old mice with colocalization analysis using the Pearson coefficient in DG and CA1. **(D–F)** Immunostaining for GFAP^+^ cells and STING antibody of young and old animals (scale bar = 100 μm, magnification: scale bar = 20 μm) followed by the Pearson coefficient analysis both in DG and CA1. Pictures were collected with a *N* = 5–7 biological replicates. All data are presented as the mean ± SEM. Symbols for *P*-values used in the figures: *P < 0.05. *P*-values were calculated using a two-tailed Student’s *t*-test.

By analyzing signals of STING and IBA1 in the hippocampus, we noticed that the numbers of STING^+^ and IBA1^+^ cells in the DG and CA1 did not match (Figures 3C, D, F, G). To determine alterations in STING expression on neurons and astrocytes in the hippocampus during aging, we performed an analysis using the Pearson coefficient to evaluate the correlation between the STING signal and the NeuN or GFAP signal. While the Pearson coefficient in microglia was constant during aging in all the regions analyzed (Supplementary Figures 3H–J), the STING protein increased its level of association with neuronal soma and astrocytes in both CA1 and DG during aging (Figure 4). It’s interesting to note that in mice aged 24 and 108 weeks, the STING signal in EC was steady across all three cell types examined, consistent with the data already shown by Western blots (Supplementary Figures 3J–L, Figure 1D).

### Autophagic deficits in the hippocampus induces STING accumulation especially in neurons

**FIGURE 5:**
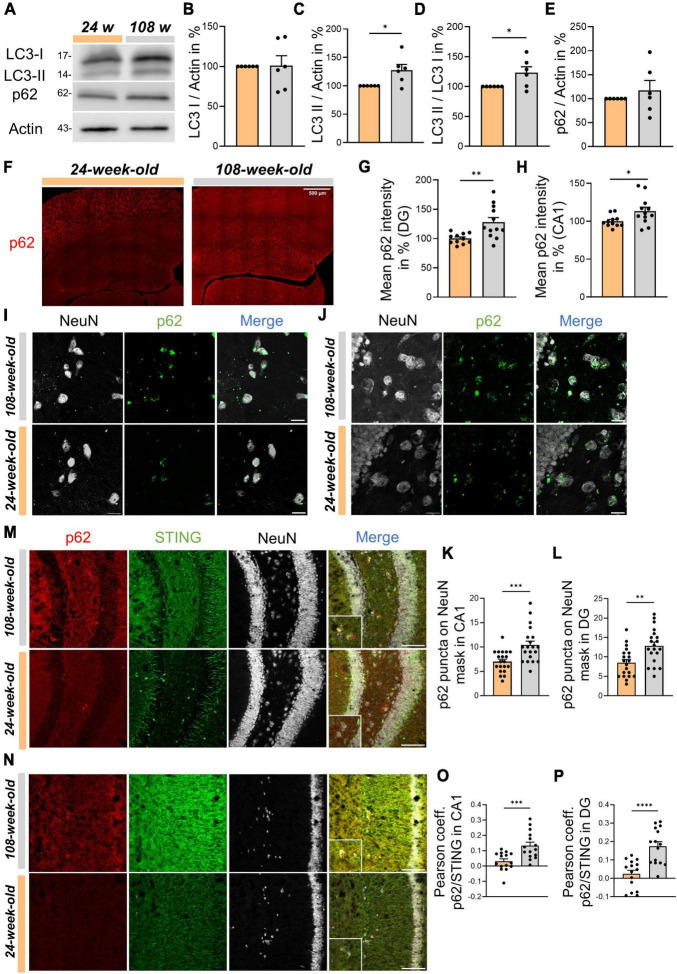
Physiological aging in mice is associated with impaired autophagy which reduces STING disposal in neurons of the hippocampus. **(A)** Representative Western blots of LC3 I, LC3 II, p62 and Actin as an internal control. **(B–E)** Statistical analysis of related protein levels from the WBs. Relative quantities were normalized to Actin. Values are the mean of three technical replicates in each group from six independent experiments for immunoblotting analysis. **(F)** Representative images of whole hippocampus sections from young and old mice immunostained with p62 (red), scale bar = 500 μm. **(G,H)** Quantification of p62 intensity in CA1 and DG from 24- to 108-week-old animals. **(I,J)** Immunostainings for p62 (green) and NeuN (white) in CA1 panel **(I)** and DG panel **(J)** with a scale bar of 20 μm and relative graphs and with associated graphs assessing the number of p62 puncta in individual neurons in different hippocampal areas, CA1 **(K)** and DG **(L)**. **(M–P)** Evaluation of Pearson coefficient between p62 and STING during hippocampal aging in CA1 panel **(M)** and DG panel **(N)** with corresponding, representative images: NeuN (white), p62 (red) and STING (green), scale bar = 100 μm. All pictures were collected from *N* = 4–6 biological replicates. All data are presented as the mean ± SEM. Symbols for *P*-values used in the figures: *P < 0.05, **P < 0.01, ***P < 0.001, ****P < 0.0001. *P*-values were calculated using a two-tailed Student’s *t*-test.

At the cellular level, STING is primarily metabolized by autophagy (Prabakaran et al., 2018; Gui et al., 2019; Liu et al., 2019; Wan et al., 2023), a process that plays a crucial role in the survival of postmitotic cells like neurons (Stavoe and Holzbaur, 2019; Valencia et al., 2021; Cai and Ganesan, 2022). In light of elevated STING signals in neuronal somata of the CA1- and DG region, we started to analyze hippocampal autophagy. First, we used p62 and LC3 as standard markers in WBs to examine autophagy. Regarding LC3, we found that 108-week-old animals accumulated significantly more LC3-II (Figures 5A–D). However, for p62 we observed a trend to increase during aging that was not statistically significant (Figure 5E). Thus, we analyzed the p62 signal’s intensity in the hippocampus using ICC. Aging significantly raised the signal intensity of p62 in both CA1 and DG (Figures 5F–H). Interestingly, we observed that in both young and old mice, the p62 signal appeared to be enhanced in NeuN^+^ spots. Next, we examined the possibility, whether aging would cause a change in the protein concentration of p62 in neurons. Therefore, we assessed the number of p62 puncta on individual neurons in the DG and CA1, using the NeuN signal as a mask. The findings indicate that p62 accumulates in the cell soma throughout hippocampal aging (Figures 5I–L). Next, we examined individual neurons in DG and CA1 to determine whether the STING signal was directly correlated with the p62 signal in order to figure out whether STING accumulation in neurons is connected to autophagy impairment. Notably, colocalization of p62 and STING in neurons was more common in the CA1 and DG of 108-week-old mice compared to young animals (Figures 5M–P). In summary, our findings suggest that an age-related autophagy impairment is directly linked to the neuronal accumulation of STING that takes place in the mouse hippocampus throughout aging.

## Discussion

In this work, alterations of cGAS-STING signaling components were analyzed in the cortex and hippocampus of 24- and 108-week-old mice. In the cortex, 108-week-old mice showed a significant increase in both cGAS protein and its major product 2′3′-cGAMP. This is in line with previous studies demonstrating that 28-month-old mice’s brains have higher 2′3′-cGAMP levels than those of younger mice (Gulen et al., 2023). Additionally, other studies have reported elevated levels of 2′3′-cGAMP in the brains of mice modeled for neurodegenerative diseases like PD in αSyn-PFF mice, AD in 5xFAD mice, and in Ataxia-Telangiectasia olfactory neurosphere-derived cells and brain organoids (Aguado et al., 2021; Hinkle et al., 2022; Xie et al., 2023). However, as we have demonstrated that TBK1 and IRF3 phosphorylation is lacking in the cortex of old mice, the increase in 2′3′-cGAMP seems to be not associated with an activation of STING. This finding goes in line with the missing gene expression of IRF3 targets analyzed, including ISG54, IFN-α, and IFN-β. We next sought to understand what causes cGAS activation in the EC during aging and found a substantial increase in dsDNA concentrations in EC neurons. Previous studies have demonstrated that cGAS activity can be triggered by DNA damage with an increase of cellular micronuclei during aging in the hippocampus, or by mitochondrial damage and the subsequent release of mtDNA into the cytosol (Aguado et al., 2021; Jiménez-Loygorri et al., 2024). Comparable results for the cortex are not published till now. Upon observing that TOM20 exhibited no alterations in the cortex with age, we directed our attention towards the identification of DNA damage. Using H2AX as a marker for damaged DNA from the nucleus we found that the DNA of neural cells from the cerebral cortex of older mice was significantly more affected, as also reported from Pao et al. (2020), Schumacher et al. (2021) and Zhao H. et al. (2022). Additionally, we discovered a marked rise of micronuclei in the EC, particularly in NeuN^+^ cells. Next, we focused on the hippocampus and analyzed the protein concentration of the key member in the cGAS-STING pathway. Thereby, we observed remarkable differences between the hippocampus and the cortex. In contrast to the cortex, hippocampus tissue does not exhibit the rise in cGAS activity during brain aging. An additional difference are the elevated levels of STING in the hippocampal regions during aging, a feature absents in the cortex. In this instance as well, we attempted to determine whether the significant rise in STING in the hippocampal region of old mice was due to increased activity of the cGAS-STING pathway. Similar to the cortex, the analysis of selected genes associated with IRF3 phosphorylation revealed no changes for both age groups. This finding is in contrast to Gulen et al., as they reported a significant increase in genes related to inflammation in 26-month-old mice (Gulen et al., 2023). Therefore, we hypothesize that brain inflammation is likely an extremely late process, manifesting between 24 (108 weeks) and 26 months of age. When analyzing the cause of the STING increase in the hippocampus, we observed a rise in the amount of microglia in the CA1 and DG areas, consistent with numerous previous reports (Choi and Won, 2011; Bachstetter et al., 2015; Barrientos et al., 2015; Gulen et al., 2023). Moreover, by analyzing other cell types, we observed that 108-week-old mice had higher STING levels compared to young mice, evaluated by Pearson coefficient on NeuN^+^ and GFAP^+^ cells. To date, it seems to be clear that STING is also expressed in neurons and astrocytes. In fact, numerous publications show that activation of the cGAS-STING pathway in astrocytes and not only in microglia is a common effect upon brain infection in mice and also in primary human astrocytes (Reinert et al., 2016; Jeffries and Marriott, 2017). STING also appears to have a role in neurons as it has been shown that neuronal lack of STING in mouse embryos causes neurogenic abnormalities and autistic-like behaviors (Zhang et al., 2020).

Several key immune regulatory factors involved in type I IFN signaling, such as cGAS and STING, are also regulated by autophagy, which in turn can affect type I IFN production (Gui et al., 2019; Wan et al., 2023). Furthermore, the cGAS-STING pathway can trigger non-canonical autophagy via LC3 and p62 (Liu et al., 2019; Sharma et al., 2020). Indeed, recent studies show that activation of STING can cause lipidation of LC3 (Prabakaran et al., 2018; Gui et al., 2019). Moreover, it has recently been shown that mouse primary neurons *in vitro* can accumulate STING following autophagosome-lysosome blockade with chloroquine treatment (Wang et al., 2024), pointing towards the fact that the autophagic mechanism is responsible for the elimination of STING (Prabakaran et al., 2018; Liu et al., 2019; Wan et al., 2023; Wang et al., 2024). In the DG and CA1 regions neurons from older mice contain significantly more p62 than young ones. Together with the age dependent rise of LC3-II in the hippocampus, this characterizes an autophagy dysfunction associated with aging as described for neurons (Moreno-Blas et al., 2019; De Risi et al., 2020; Cai and Ganesan, 2022). Additionally, the observed autophagic malfunction seems to be responsible for the STING accumulation in autophagosomes assessed by colocalization analysis between STING and p62 in NeuN^+^ areas of CA1 and DG.

These results have several limitations and this work is preliminary in many aspects.

Firstly, the information provided are merely descriptive and gives an overview of what we discovered by brain analysis of 24- and 108-week-old mice. Additionally, it could be that the 108-week-old group is still too young to develop inflammation, as previous studies have demonstrated increasing inflammation in the brain only in 28-month-old mice (Gulen et al., 2023). In this article, we mainly described the putative role of cGAS-STING pathway in EC and hippocampus during aging. Probably, this signaling has a different influence in other brain areas as well. Finally, for these experiments we used animals that consisted of an equal number of males and females. Since aging is a different phenomenon between men and women, it would be interesting to evaluate the differences that the cGAS-STING pathway may have between the different sexes. It is plausible, nevertheless, that the cGAS-STING pathway could contribute to brain aging in a gender-specific manner.

In summary, this study’s findings show that, depending on the region analyzed, the cGAS-STING pathway’s activity varies with normal brain aging. We found that cGAS activity is enhanced by the presence of micronuclei and DNA damage in neurons of the cortex. Conversely, we neither did observe increasing cGAS activity nor an increase in dsDNA or H2AX in the hippocampal region, but we found impaired neuronal function associated with a deficiency in autophagy that led to the accumulation of STING. A deeper comprehension of the function of the cGAS-STING pathway throughout aging and whether its potential manipulation could aid in delaying senescence or curing neurodegenerative diseases may be conceivable with more focused research on specific brain regions.

## Data availability statement

The data are available from the corresponding author upon reasonable request.

## Ethics statement

The animal study was approved by the Ethics Commission of the Federal State of Saxony-Anhalt (42502-2-1507 Uni MD and 42502-2-1578 Uni MD). The study was conducted in accordance with the local legislation and institutional requirements.

## Author contributions

SP: Writing−review and editing, Writing−original draft, Visualization, Validation, Methodology, Investigation, Formal analysis, Data curation, Conceptualization. SK: Writing−review and editing, Visualization, Validation, Investigation, Data curation. KB: Writing−review and editing, Methodology, Investigation. I-CT: Writing−review and editing, Formal analysis, Data curation. KC: Writing−review and editing, Methodology, Investigation. AK: Writing−review and editing, Supervision, Resources, Conceptualization. DD: Writing−review and editing, Supervision, Resources, Funding acquisition, Conceptualization. PL: Writing−review and editing, Writing−original draft, Visualization, Validation, Supervision, Data curation, Conceptualization.
