# Supplementary material for: Alteration of cGAS-STING signaling pathway components in the mouse cortex and hippocampus during healthy brain aging
Source: Front Aging Neurosci. 2024 Aug 1;16:1429005. doi: 10.3389/fnagi.2024.1429005 (PMC11324507; doi:10.3389/fnagi.2024.1429005)
Supplement: Supplementary file 1 [file Data_Sheet_1.docx]

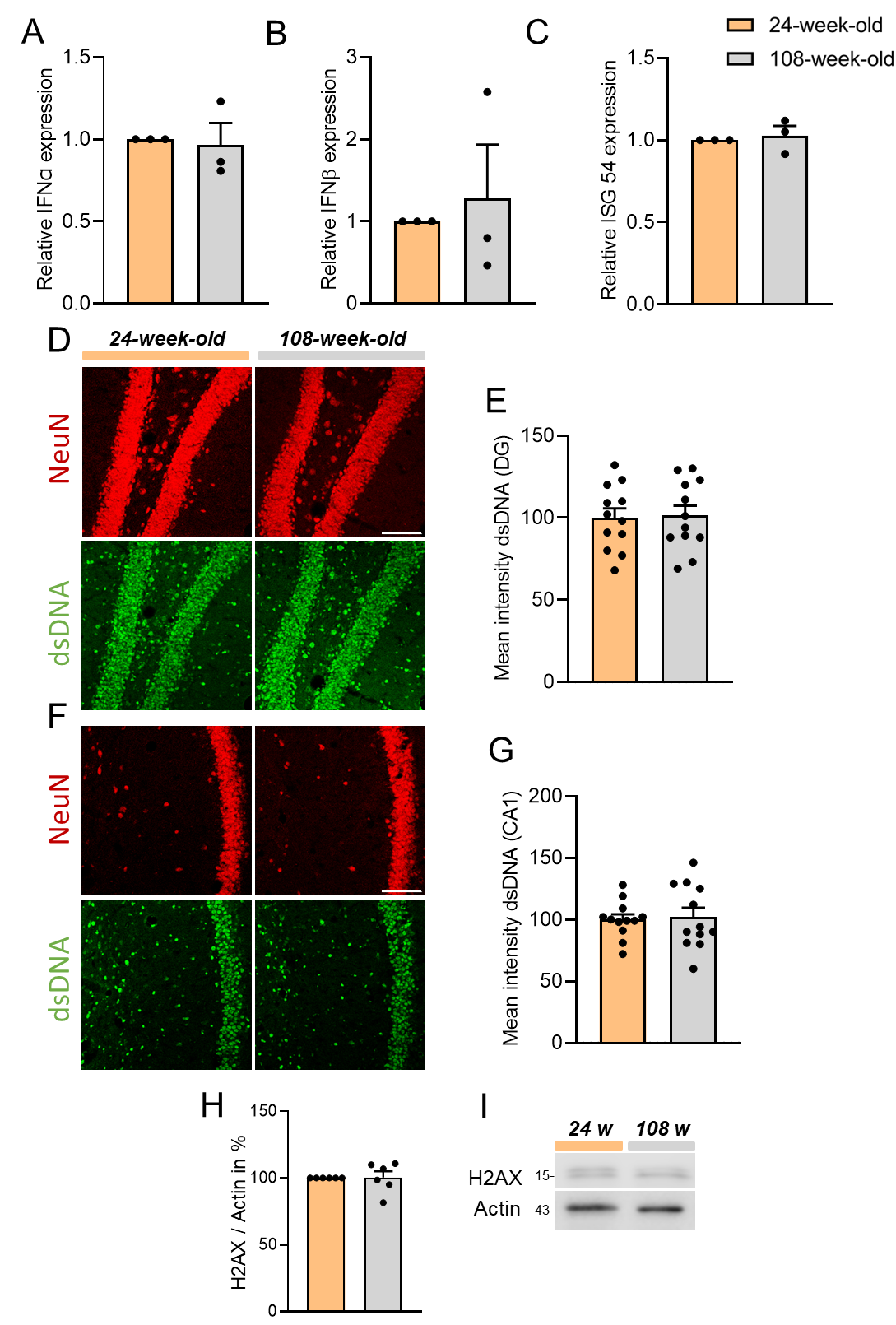


***Supplementary figure 1. Expression analysis of genes involved in inflammation from cortex tissue and amount of dsDNA in DG and CA1 of the hippocampus.*** A,B,C, Gene expression of IFN-α, and IFN-β, ISG54 from cortical tissues of young and aged mice (N=3). D,E,F,G, Representative images and quantification of the intensity of dsDNA in the DG (D,E) and CA1 (F,G) from 24- and 108-week-old mice. H,I Immunoblot results with representative blots of H2AX on hippocampal tissue of young and old animals. N=3 biological replicates. All data are presented as the mean ± SEM. *P* values were calculated using a two-tailed Student’s t test.


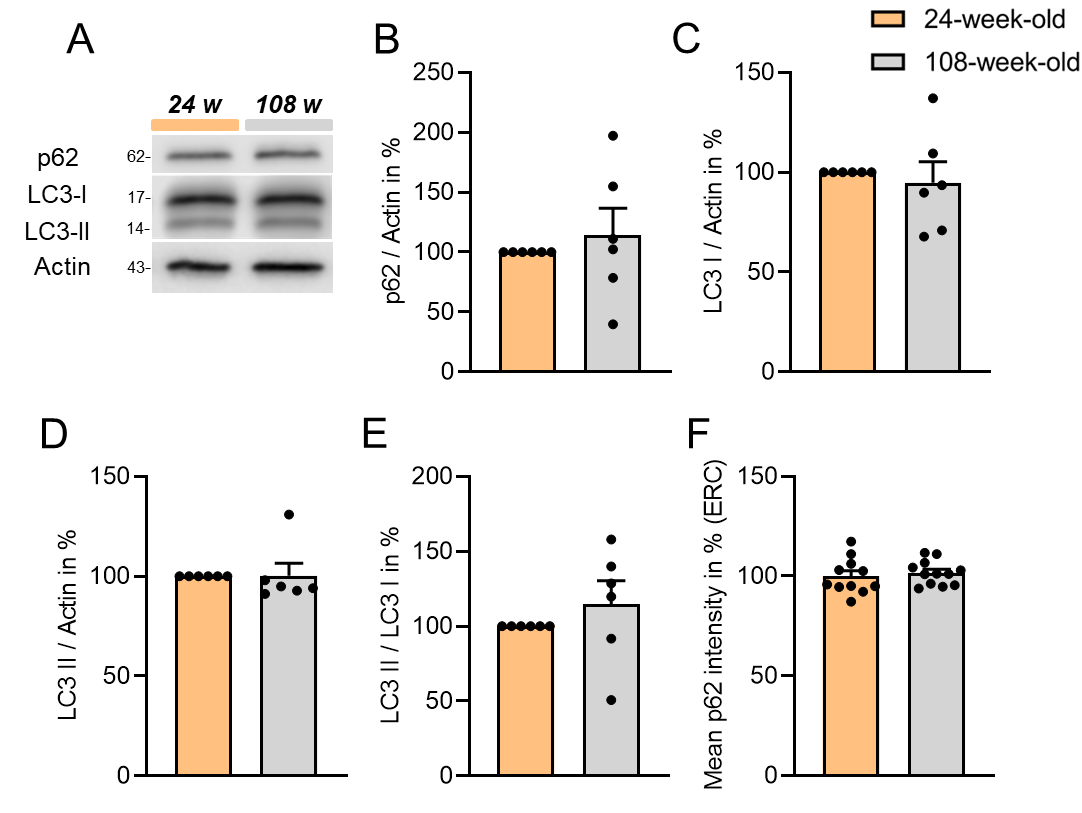


***Supplementary figure 2. Analysis of the principal autophagic markers via WB and IHC on cortex of young and old mice.*** A-E, Representative blots of p62 and LC3 proteins and Actin as an internal control with correlated determination of protein levels during cortical aging. Relative quantities were normalized to Actin. Values are the mean of three technical replicates in each group from six independent experiments for immunoblotting analysis. F, Evaluation of the intensity of p62 in the EC region during brain aging. All data are presented as the mean ± SEM*. P* values were calculated using a two-tailed Student’s t test.


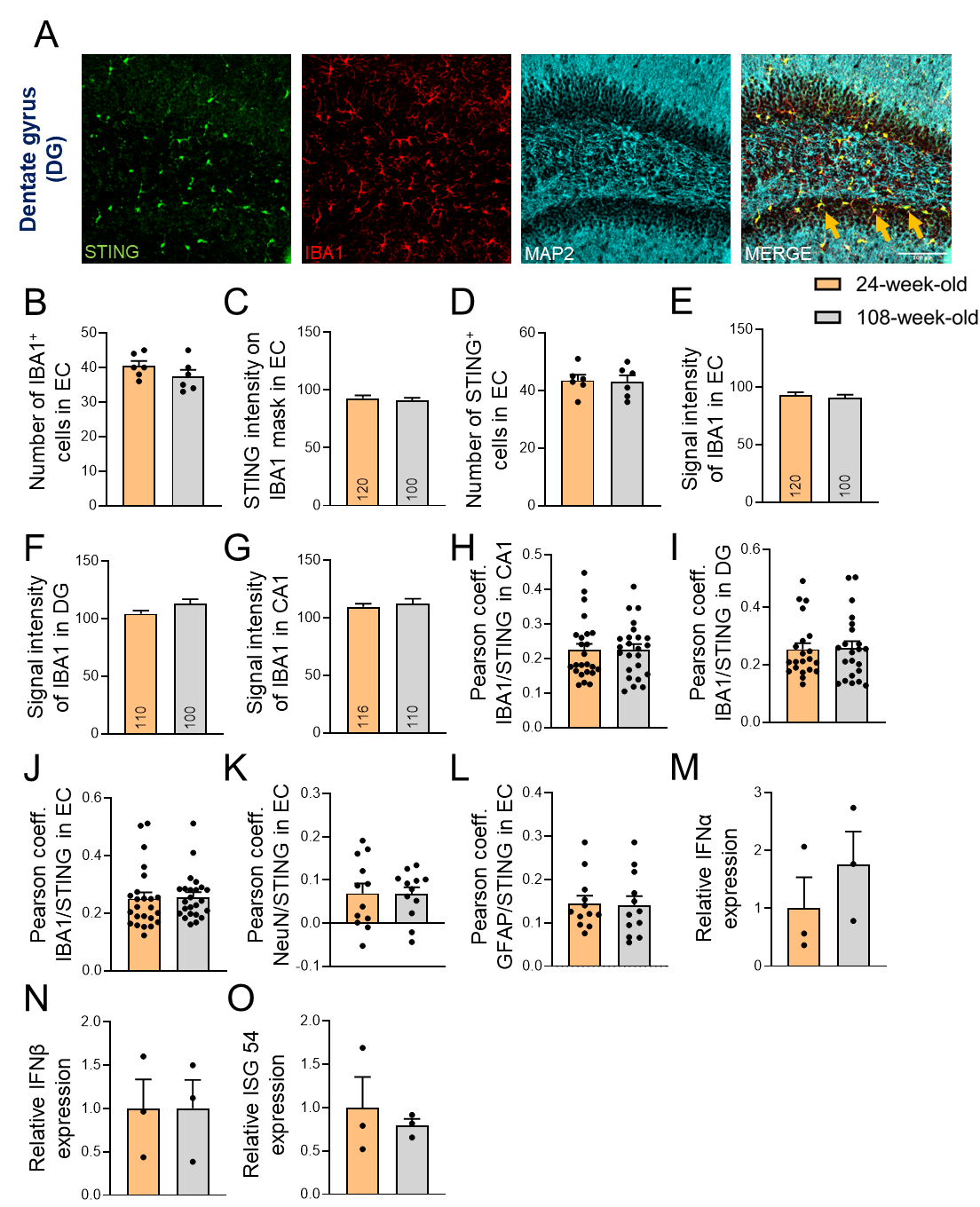


***Supplementary figure 3.*** ***IBA1-STING co-staining and analysis of the signals in CA1 and DG of the hippocampus and EC.*** A, Frontal brain slice were immunohistochemically stained using antibodies raised against STING (green), IBA1 (red) and MAP2 (cyan). Representation of dentate gyrus, showing strong overlap between STING and IBA1 positive microglia cells. Scale bar = 100 µm. B-E, Evaluation of number of IBA1^+^ and STING^+^ cell and intensity of IBA1 and STING on a IBA1 mask in EC. F,G, Assessment of signal intensity of IBA1 in hippocampal region like CA1 and DG. H-J, Graphs representing the colocalization express in Pearson coefficients between IBA1 and STING in CA1, DG, and EC. K,L, Pearson coefficients of NeuN/STING and GFAP/STING in EC. Values regarding evaluation of signal intensity, number of IBA1^+^ and STING^+^ cells, and Pearson coefficient were obtained with at least three technical replicates in each group from four to six independent experiments. M-O, Gene expression of IFN-α, and IFN-β, ISG54 from hippocampal tissues of young and aged mice (N=3). All data are presented as the mean ± SEM. *P* values were calculated using a two-tailed Student’s t test.
